# Bevacizumab and Breast Cancer: A Meta-Analysis of First-Line Phase III Studies and a Critical Reappraisal of Available Evidence

**DOI:** 10.1155/2012/417673

**Published:** 2012-09-12

**Authors:** José R. Rossari, Otto Metzger-Filho, Marianne Paesmans, Kamal S. Saini, Alessandra Gennari, Evandro de Azambuja, Martine Piccart-Gebhart

**Affiliations:** ^1^Institut Jules Bordet, Université Libre de Bruxelles, Boulevard de Waterloo, 125, 1000 Brussels, Belgium; ^2^Medical Oncology, Galliera Hospital, Via Volta 6, 16125 Genoa, Italy

## Abstract

*Background*. Randomized studies have shown different magnitude of bevacizumab benefit in the treatment of advanced breast cancer. Regulatory agencies have modified bevacizumab treatment indications across different regions. In this study, we perform a meta-analysis of phase III studies aiming to interrogate the magnitude of bevacizumab benefit for the treatment of first-line HER2-negative metastatic breast cancer (MBC). *Methods*. Data from studies E2100, AVADO and RIBBON-1 were used to calculate the benefit of bevacizumab in terms of tumor overall response rate (ORR), progression-free survival (PFS), overall survival (OS), and toxicities. Combined statistical estimates of hazard ratios (HR) and odds ratios were calculated using fixed-effects or random-effects models. *Results*. A total of 2,695 patients were evaluated. Combining bevacizumab with different chemotherapy backbones resulted in a 30% risk reduction of PFS events (HR = 0.70; 95% confidence interval [CI], 0.57–0.86) and increased ORR (odds ratio 1.81; 95% CI, 1.53–2.14). No OS benefit could be demonstrated (HR = 0.95; 95% CI, 0.85–1.06). Bevacizumab significantly increased the incidence of adverse events such as proteinuria, hypertension and cardiovascular events. *Conclusions*. Bevacizumab combined with chemotherapy in the first-line treatment of MBC significantly improved ORR and PFS, but also increased grade 3-4 toxicities. No significant OS advantage was observed.

## 1. Introduction

Vascular endothelial growth factor (VEGF) and its receptors are thought to play a pivotal role in tumor angiogenesis [1]. Bevacizumab is a humanized monoclonal antibody designed to block VEGF-A and has proved to be effective in colorectal cancer, nonsmall cell lung cancer, renal cell carcinoma, ovarian carcinoma, and glioblastoma multiforme [2–8].

In the field of breast cancer, bevacizumab has generated more controversies and discussions than any other targeted therapy. In February 2008, the FDA granted accelerated approval to bevacizumab in combination with paclitaxel for the first-line treatment of metastatic, HER2-negative metastatic breast cancer (MBC), based on promising results of the Eastern Cooperative Oncology Group (ECOG) 2100 trial. On July 20th 2010, the Oncologic Drugs Advisory Committee (ODAC) of the FDA's Center for Drug Evaluation and Research voted 12 to 1 against the use of bevacizumab in combination with chemotherapy for the first-line treatment of advanced breast cancer [9]. This was followed by a definitive announcement by the FDA revoking approval of bevacizumab for this indication [10].

Nonetheless bevacizumab is still approved by different regulatory agencies across several countries as a standard antiangiogenic drug for the treatment of first-line advanced breast cancer. With more than 20,000 breast cancer patients currently being randomized into bevacizumab studies, it is crucial to define which magnitude of endpoint or risk/benefit ratio is expected. In this study, we performed a meta-analysis of randomized phase III studies evaluating bevacizumab for the first-line treatment of metastatic breast cancer [11–13]. The magnitude of risks and benefits of adding bevacizumab to the standard treatment of advanced cancer are discussed in the context of recent controversies and ongoing randomized phase III clinical trials.

## 2. Methods

### 2.1. Study Selection and Data Extraction

MEDLINE searches were performed to identify eligible studies, which were restricted to phase III, randomized, controlled trials comparing the combination of bevacizumab to chemotherapy with chemotherapy alone for the first-line treatment of mostly HER2-negative advanced breast cancer. The proceedings of the San Antonio Breast Cancer Symposium, European Society of Medical Oncology, and American Society of Clinical Oncology annual meetings were examined for presented abstracts. Based on these criteria, the E2100, AVADO, and RIBBON-1 were selected for our meta-analysis.

### 2.2. Data Extraction

Data abstraction was conducted independently by three investigators (J. R. Rossari, O. Metzger-Filho, and M. Paesmans) in accordance with the Preferred Reporting Items for Systematic Reviews and Meta-Analyses (PRISMA) guidance [14]. For each study the following information was extracted: publication or presentation date, first author's last name, sample size, primary endpoints, regimens used, dosage and scheduling of chemotherapy and bevacizumab, line of treatment, number of chemotherapy cycles, additional treatments given (regardless of study arm), follow-up period, number of outcome events, information pertaining to study design, PFS definition, tumor response criteria, data on PFS, ORR and OS, subgroup evaluation, quality of life analysis, crossover, if any, and toxicities. 

### 2.3. Statistical Methods

The impact on PFS and OS of adding bevacizumab to a chemotherapy regimen was measured in terms of the hazard ratio (HR). For each study, the HR was either extracted directly from the reports, or it was estimated. Two of the studies included in this meta-analysis were designed to compare more than one bevacizumab arm with a control group, either to evaluate different doses of the drug (AVADO), or to evaluate its combination with different cytotoxic agents (RIBBON-1). To render the statistical analysis of these comparisons feasible, each analysis was treated as a different study, and a HR was extracted from all of them. Whenever possible, HR was also estimated for PFS according to subgroups of patients defined a priori (age, hormone receptor status, prior adjuvant chemotherapy, prior taxane therapy, and length of disease-free interval [DFI]). For some subgroups, it was necessary to extrapolate HRs and their variances from graphical representations [15]. Individual HR estimates were then combined into overall HR using fixed effects or random effects models, depending on the outcome of the heterogeneity *X* ^2^-test. If heterogeneity was not detected (at the 10% significance level), the fixed-effects model was applied. Heterogeneity was quantified by the *I* ^2^ coefficient that measures the percentage of total variation across studies that is due to heterogeneity rather than chance [16]. By convention, a HR < 1.00 implied a benefit of adding bevacizumab to a chemotherapy regimen. This impact was considered statistically significant if the 95% confidence interval (CI) for overall HR did not overlap 1.00 [17].

The association of bevacizumab with toxicities and response rate were calculated in terms of odds ratios, applying the same statistical methods described above. 

## 3. Results

### 3.1. First-Line Bevacizumab Studies in Metastatic Breast Cancer

Three randomized phase III studies evaluating the impact of adding bevacizumab to chemotherapy in the first-line treatment of HER2-negative MBC have reported positive results in terms of overall response rate (ORR) and progression-free survival (PFS). E2100, AVADO, and RIBBON-1 are the focus of this meta-analysis and the key results of the three trials are summarized in Table 1 [11–13]. The E2100 study showed that the addition of biweekly bevacizumab 10 mg/kg to weekly paclitaxel doubled the PFS when compared with paclitaxel alone in the first-line treatment of patients with HER2-negative MBC without an overall survival (OS) gain [11]. Treatment was continued until unacceptable toxicity or disease progression and no crossover was allowed. Subsequently, the AVADO study randomized patients to docetaxel alone or in combination to bevacizumab (at two dose levels of 15 mg/kg or 7.5 mg/kg) and demonstrated improvement in PFS and no OS benefit. In the AVADO study treatment was continued for up to nine weeks, disease progression, or unacceptable toxicity. In contrast to E2100, 40% of patients enrolled on AVADO received bevacizumab in the second-line setting. The RIBBON-1 trial in which bevacizumab 15 mg/kg was added to capecitabine, anthracycline, or taxane-based chemotherapy similarly showed improved PFS without OS benefit [13]. Approximately 60% of patients randomized in the RIBBON-1 trial received bevacizumab as second-line chemotherapy. The PFS gain observed in the E2100 trial was not replicated with the same magnitude in the subsequent phase III studies (AVADO and RIBBON-1), and OS could not be demonstrated.

The three studies selected for this meta-analysis represented a total of 2,695 patients, with the number of patients in each study ranging from 722 to 1,237. The AVADO and RIBBON-1 trials were double-blinded, placebo-controlled studies, while the E2100 trial was an open-labeled study with patients randomly assigned to paclitaxel alone or the combination of paclitaxel and bevacizumab. Median follow-up times were provided only in AVADO and RIBBON-1 studies.

### 3.2. Combined Analysis of First Line Studies (E2100, AVADO, and Ribbon-1)

#### 3.2.1. Progression-Free Survival

**Figure 1:**
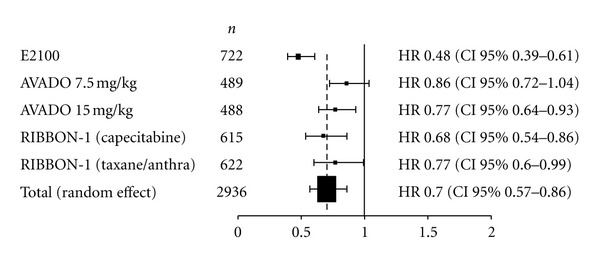
Progression-free survival hazard ratios. Abbreviations: Anthra: anthracycline; *n*: number of patients.

Figure 1 shows the HR for PFS in each individual trial and the overall analysis. The HR for PFS of the bevacizumab arms were evaluated separately and compared to the control arms in the AVADO and RIBBON-1 trials. Individually, a consistent PFS benefit was observed across all three trials. Our meta-analysis shows a statistically significant benefit obtained by adding bevacizumab to chemotherapy in the first-line treatment of MBC patients: the overall HR was 0.70 (95% CI, 0.57 to 0.86), corresponding to a 30% reduction of the hazard of progression for bevacizumab-based regimens. Statistically significant heterogeneity was observed between the studies (*P* = 0.0006), *I* ^2^ = 77%.

#### 3.2.2. Progression-Free Survival according to Subgroups

PFS was assessed according to hormone receptor status (positive or negative), prior adjuvant chemotherapy (yes or no), age (<65 years versus ≥65 years), use of prior taxane (yes or no), and DFI (short or long), although the definition of DFI differed between the trials. For instance, AVADO and E2100 trials stratified DFI in ≤24 months versus >24 months, while RIBBON-1 considered ≤12 months versus >12 months.

**Figure 2:**
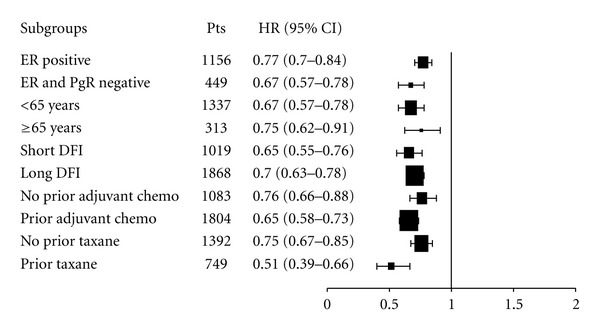
Progression-free survival hazard ratios across subgroups. Abbreviations: chemo: chemotherapy, DFI: disease-free interval, ER: estrogen receptor, PgR: progesterone receptor.

The addition of bevacizumab to chemotherapy consistently showed a PFS benefit in all analyzed subgroups, as shown in Figure 2. Interaction tests were carried out and did not reveal any significant interaction between analyzed covariates and bevacizumab effect (*P* = 0.74).

#### 3.2.3. Overall Survival

**Figure 3:**
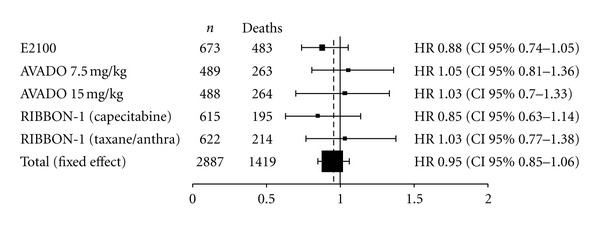
Overall survival hazard ratios. Abbreviations: Anthra: anthracycline, *n*: number of patients.

Figure 3 shows the HR for OS in each individual trial and the overall analysis. As was the case with PFS, the HRs of the clinical trials with two bevacizumab arms (AVADO and RIBBON-1) were evaluated separately, and each was compared to the control group. Individually, none of the studies showed a significant OS benefit of adding bevacizumab to chemotherapy as first-line treatment of MBC. Our results show no statistically significant benefit of adding bevacizumab to chemotherapy in the first-line treatment of MBC patients (HR 0.95, 95% CI, 0.85 to 1.06). No heterogeneity was identified between the trials (*P* = 0.65), *I* ^2^ = 0%. 

#### 3.2.4. Overall Response Rate

**Figure 4:**
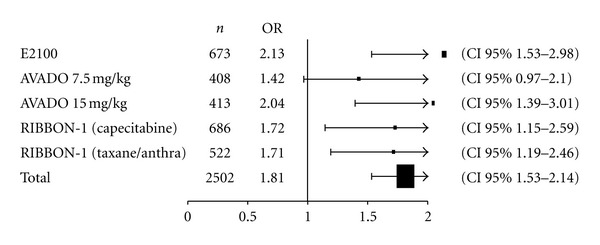
Overall response rate (ORR) Odds Ratio (OR). Abbreviations: Anthra, anthracycline; *n*, number of patients.

As shown in Figure 4, the odds ratio of response associated with the addition of bevacizumab to chemotherapy was 1.81 (95% CI, 1.53–2.14). Again, no heterogeneity was seen between trials (*P* = 0.55), *I* ^2^ = 0%. The results did not change when the arm of the RIBBON-1 trial containing capecitabine (and no taxane) was removed from the analysis (odds ratio 1.83; 95% CI, 1.52–2.19; *P * test for heterogeneity 0.39) to evaluate the effect of adding bevacizumab to a taxane-based chemotherapy.

#### 3.2.5. Safety Profile of Bevacizumab

The addition of bevacizumab to chemotherapy increased the probability of grade 3-4 hypertension (random effects odds ratio 5.56; 95% CI, 1.66–18.62), proteinuria (fixed effects odds ratio 5.35; 95% CI, 2.80–10.20), sensory neuropathy (fixed effects odds ratio 1.48; 95% CI, 1.11–1.99), and cardiac events including left ventricular (LV) dysfunction and congestive heart failure (fixed effects odds ratio 3.36; 95% CI, 1.41–8.01). No significant increase in the risk of gastrointestinal (GI) perforation was seen in MBC patients treated with bevacizumab (fixed effects odds ratio 0.94; 95% CI, 0.31–2.85) [18].

## 4. Discussion

Bevacizumab combined with chemotherapy in the first-line treatment of MBC significantly improved ORR and PFS, but also increased grade 3-4 toxicities. No significant OS advantage was observed. One of the most frequently cited reasons for conducting a meta-analysis is the increase in statistical power that it affords; however, inherent limitations may limit the accuracy of results. In this study, we acknowledge the following limitations: First, it was conducted using published study results, rather than individual patient data. Second, for the two studies with more than one bevacizumab containing-arm, control arms had to be duplicated in order to give each comparison independent statistical treatment. Third, despite being the primary endpoint in all three trials, the definition of PFS was not precisely specified in all of them. Despite limitations our results provided similar conclusions when compared to other studies [19–22]. Three meta-analyses evaluated the efficacy of bevacizumab plus chemotherapy for the treatment of MBC, and provide interesting points of comparison with our study [19–21]. Valachis et al. analyzed five studies, including a phase II study and one trial with bevacizumab and capecitabine after first-line chemotherapy for MBC, and found global HRs similar to the ones we report: 0.70 (95% CI 0.60–0.82) for PFS and 0.90 (95% CI 0.80–1.03) for OS [19]. Lee et al. analyzed four studies involving a total of 2,860 patients, to verify the clinical efficacy of bevacizumab in the salvage treatment of MBC, and reported PFS (HR 0.69, 95% CI, 0.58–0.81), OS (HR 0.92, 95% CI, 0.82–1.03), and ORR (HR 1.53, 95% CI, 1.37–1.71) [20]. O' Shaughnessy et al. conducted a meta-analysis including individual patient data from the E2100, AVADO, and RIBBON-1 studies and showed a 36% reduction in the risk of a PFS event (HR = 0.64, 95% CI 0.57–0.71) and no median OS gain (HR = 0.97; 95% CI 0.86–13.08) [21]. However, one-year survival rate was statistically significant increased for patients treated in the bevacizumab-arms (77% versus 82%, *P* = 0.003) [21]. 

In this study, the addition of bevacizumab to chemotherapy statistically increased PFS in all analyzed subgroups. Importantly, HER2-positive disease was not allowed in the AVADO and RIBBON-1 studies, and only about 1% of patients participating in E2100 had HER2 overexpressing tumors. Our results seem to concur with those obtained by O'Shaughnessy et al. in a comparison of the subgroup analyses performed in the same studies [23]. Similar PFS benefit with the addition of bevacizumab was observed in patients according to hormone receptor-negative status, age, and previous exposure to taxane. Comparing with their meta-analysis, beyond efficacy in terms of PFS and OS, our study also adds relevant information about the safety profile of bevacizumab in this population of patients.

The results of individual bevacizumab phase III studies and meta-analyses motivated important discussions about adequate endpoints for breast cancer studies. Whether PFS benefit can be used as a surrogate for OS in breast cancer patients is a matter of debate, and OS continues to be the endpoint of choice to assess the efficacy of new treatments for patients with MBC [24]. The availability of bevacizumab as second-line treatment for some patients enrolled in the AVADO and RIBBON-1 studies might therefore be considered a confounding factor, which could in turn have affected the chance of demonstrating an OS benefit in the intent-to-treat analysis. 

An earlier phase III study failed to demonstrate PFS benefit when bevacizumab was added to capecitabine for patients with MBC previously treated with both an anthracycline and a taxane, and at least one prior chemotherapy regimen for metastatic disease [25]. That finding has been recently challenged by the results of the RIBBON-2 trial, which demonstrated ORR and PFS gains with the addition of bevacizumab to second-line chemotherapy [26]. If the benefits of bevacizumab are extended to subsequent lines of treatment after first disease progression, crossover is likely to pose an important obstacle for detecting OS gains in first-line chemotherapy.

Recently, the results of two large neoadjuvant clinical trials evaluating the addition of bevacizumab to different chemotherapy regimens further increased the controversies surrounding bevacizumab therapy for breast cancer [10]. Both the National Surgical Adjuvant Breast and Bowel Project (NSABP) B-40 [27] and the GeparQuinto (GBG44) [28] were designed to evaluate whether the addition of bevacizumab to chemotherapy would increase the rates of Pathologic Complete Response (PCR) in women with early-stage HER2-negative breast cancer. In both studies, the rates of PCR defined as the absence of invasive disease in the breast, irrespective of nodes, were in favor of bevacizumab (16.5% without bevacizumab versus 20.5% with bevacizumab in the GBG44 trial, and 28.2% versus 34.5% in the NSABP B-40 trial). However, neither trial showed significant differences when PCR was defined as the absence of invasive disease in the breast and lymph nodes (GBG44: 18.3% versus 21.7%, *P* = 0.07; NSABP B-40: 23.0% vresus 27.6%, *P* = 0.08, both resp. without and with bevacizumab). In contrast to our results and previous findings [23], subgroup analysis of the NSABP B-40 trial and GBG44 studies suggested differential benefit of bevacizumab according to breast cancer subtypes [27, 28]. Intriguingly, the results were not in the same direction. In the GBG44, the rates of PCR were increased with bevacizumab in patients with hormone receptor-negative, while the NSABP B-40 showed increased PCR in the with hormone receptor-positive cancers. 

In the current analysis, bevacizumab therapy was associated with increased proteinuria, hypertension, cardiovascular dysfunction, and sensory neuropathy. In agreement with our findings, a combined analysis of five phase III trials in advanced breast cancer showed a statistically significant increase in proteinuria (OR = 27.68), hypertension (OR = 12.76), and left ventricular dysfunction (LVD) (OR = 2.25) with the addition of bevacizumab [29]. In addition, hemorrhagic events (OR = 4.07) were also associated to bevacizumab [29]. Moreover, several meta-analyses have been reported linking specific adverse events to bevacizumab therapy. Gastrointestinal perforation was associated to bevacizumab therapy among 12,294 patients evaluated with a relative risk (RR) of 2.14 (95% CI 1.19–3.85), but statistically significant only for colorectal cancer patients [18]. Hypertension was associated to bevacizumab therapy in a meta-analysis conducted among 12,049 patients across several tumor subtypes with a RR of 5.38 (95% CI 3.63–7.97) [30]. Venous thromboembolism was associated to bevacizumab therapy among 7,956 patients studied with a RR of 1.33 (95% CI 1.13–1.56), but not significant in the breast cancer subset [31]. Arterial thromboembolic events including cardiac ischemia was increased in a meta-analysis including 12,617 patients with a RR of 1.44 (95% CI 1.08–1.91). An additional study found an incidence of arterial thromboembolism of 0.7% in BC patients treated with bevacizumab, which was not significantly higher when compared to chemotherapy alone (RR = 1.47, 95% CI 09.27–7.95) [32]. Serious congestive heart failure (CHF) was associated to bevacizumab therapy among 3,784 BC patients evaluated with a RR of 4.74 (95% CI 1.66–11.18) [33]. A higher risk of fatal adverse event was associated with bevacizumab therapy among 10,217 patients evaluated (RR = 1.46, 95% CI, 1.09–1.94). However, in the subset of breast cancer studies no significant increase in fatal adverse events was observed (RR = 0.69, 95% CI, 0.3–1.62) [34]. Fatal adverse events were attributed mainly to gastrointestinal perforation, neutropenia, and hemorrhage and more likely to occur among patients with pancreatic and lung cancer. For instance, in the Athena trial, which prospectively evaluated the safety of bevacizumab in combination to taxane regimens among 2,551 BC patients [35], fatal adverse events occurred in 0.7% of patients. 

Hence, a clear understanding of the magnitude of bevacizumab benefit, toxicity, and benefit across breast cancer subgroups is of paramount importance. Bevacizumab is under evaluation across several phase III studies and correct estimates of treatment related toxicities and efficacy from previous studies are fundamental to the appropriate guidance and conduct of ongoing studies (Table 2). In the advanced setting, phase III studies are estimated to enroll over 4,000 patients. Bevacizumab is being studied in combination to different chemotherapy regimens (NCT00600340, NCT01303679, NCT01131195, NCT00785291); as maintenance therapy (NCT01250379, NCT00929240); in combination with more than one cytotoxic drug (NCT01200212); in combination with trastuzumab (NCT00520975, NCT00391092); and in combination with hormonal therapy. Cancer and Leukemia Group B (CALGB) 40503 trial (NCT00601900) randomizes patient with locally advanced or metastatic BC to receive tamoxifen or letrozole, with or without bevacizumab. Similarly, another study (NCT00545077) randomizes postmenopausal patients with advanced BC for endocrine therapy (letrozole or fulvestrant) alone or in combination with bevacizumab. Importantly, a broad spectrum of phase III studies are expected to randomize about 15,000 early breast cancer patients into four large adjuvant studies (Table 3).

The recent decisions by FDA regarding use of bevacizumab in patients with MBC has turned the spotlight on the risk-versus-benefit of adding bevacizumab to chemotherapy [36]. Today, thousands of women with breast cancer are being randomized into bevacizumab studies; therefore, it is imperative to define which magnitude of endpoint or risk/benefit ratio is expected. Moreover, there is a great need to identify and validate biomarkers to aid clinical decisions in the treatment with antiangiogenic therapies. The side effects associated with bevacizumab are considerable and predictive biomarkers to identify subgroups most likely to benefit are needed. In a retrospective analysis conducted in the E2100 study, VEFG polymorphisms were able to predict not only bevacizumab benefit but also toxicity [37]. However, validation of biomarker findings in subsequent studies is needed and the incorporation of translational research questions into prospective clinical trials should be mandatory.

## Figures and Tables

**Table 1:** Phase III studies with bevacizumab and chemotherapy as first-line treatment of metastatic breast cancer.

Study	Treatment line	Arms	Patients	Response rate	Progression-free survival	Overall survival	Crossover
E2100 (2007)	First	Paclitaxel q1w +/− Bev 10 mg/kg q2w	722	**36.9%** versus **21.2%** (*P* < 0.001)	**11.8** versus **5.9** months [HR 0.6 (0.51–0.7)]	**26.7** versus **25.2** months [HR 0.88 (*P* = 0.16)]	Not allowed

AVADO (2008)	First	Docetaxel q3w + Bev 15 mg/kg or Bev 7.5 mg/kg or Placebo q3w	736	**64%** (*P* < 0.001) versus **55%** (*P* = 0.07) versus **46%**	**10.1** [HR 0.77 (0.64–0.93)] versus **9.0** [HR 0.86 (0.72–1.04)] versus **8.2** months	**30.2** [HR 1.03 (0.7–1.3)] versus **30.8** [HR 1.05 (0.81–1.36)] versus **31.9** months	Allowed

RIBBON 1 (2009)	First	Capecitabine q3w + Bev 15 mg/kg q3w or Placebo q3w Anthracycline^1^/ Taxane^2^ q3w + Bev 15 mg/kg q3w or Placebo q3w	1,237	**35.4%** versus **23.6%** (*P* = 0.009) **51.3%** versus **37.9%** (*P* = 0.005)	**8.6** versus **5.7** months [HR 0.69 (0.56–0.84)] **9.2** versus **8.0** months [HR 0.64 (0.52–0.80)]	**29.0** versus **21.2** months [HR 0.85 (0.63–1.14)] **25.2** versus **23.8** months [HR 1.03 (0.77–1.38)]	Allowed

^1^Adriamycin or Epirubicin + Cyclophosphamide +/− 5-Fluorouracil q3w; ^2^Docetaxel or nab-Paclitaxel q3w.

Abbreviations: ORR: overall response rate; PFS: progression-free survival; HR: hazard ratio; LD: low dose; HD: high dose.

**Table 2:** Ongoing a phase III clinical trials evaluating the addition of bevacizumab for the treatment of advanced breast cancer.

Identifier	Setting	*n*	Study Design
NCT00600340	Advanced	560	Bevacizumab + paclitaxel versus bevacizumab + capecitabine
NCT01303679	Advanced	198	Bevacizumab + exemestane versus bevacizumab + paclitaxel
NCT01131195	Advanced	142	Bevacizumab + paclitaxel versus metronomic ctx + capecitabine
NCT01250379	Advanced	488	Chemotherapy + bevacizumab. versus chemotherapy*
NCT00929240	Advanced		Bevacizumab + capecitabine. versus bevacizumab**
NCT00785291	Advanced	900	Bevacizumab + paclitaxel versus paclitaxel Bevacizumab + nab-paclitaxel versus nab-paclitaxel Bevacizumab + ixabepilone versus ixabepilone
NCT01200212	Advanced	432	Bevacizumab + taxane + capecitabine versus taxane + capecitabine
NCT00545077	Advanced	378	Letrozole or fulvestrant versus letrozole or fulvestrant + bevacizumab
NCT00601900	Advanced	502	Tamoxifen or letrozole versus tamoxifen or letrozole + bevacizumab
NCT00391092	Advanced	407	Bevacizumab + trastuzumab + docetaxel versus trastuzumab + docetaxel
NCT00520975	Advanced	489	Bevacizumab + trastuzumab + carboplatin + paclitaxel versus trastuzumab + carboplatin + paclitaxel

Identifiers are from clinicaltrials.gov website. *Patients previously treated with bevacizumab. **Patients treated with bevacizumab and docetaxel and no evidence of progressive disease.

Abbreviations: *n*: estimated number of patients.

**Table 3:** Ongoing adjuvant phase III trials evaluating the addition of bevacizumab for the treatment of early breast cancer.

Identifier	Study	*N*	Study population	Study design
NCT00528567	BEATRICE	2,583	Triple negative BC	Standard CT versus Standard CT + BEV for 1 year

NCT00887536	NSABP B-46	3,900	HER2 negative N+ or high risk N−	Docetaxel/cyclophosphamide × 6 versus Docetaxel/doxorubicin/cyclophosphamide × 6 versus Docetaxel/cyclophosphamide/BEV × 6 (followed by BEV alone until 1 year)

NCT00625898	BETH	3,509	HER2 positive N+ or high risk N−	Docetaxel/carboplatin/TRAST × 6 (followed by TRAST alone until 1 year) versus Docetaxel/carboplatin/TRAST/BEV × 6 (followed by TRAST/BEV until 1 year) versus Docetaxel [×3]/FEC [×3]/TRAST × 6 (followed by TRAST alone until 1 year) versus Docetaxel [×3]/FEC [×3]/TRAST/BEV × 6 (followed by TRAST/BEV until 1 year)

NCT00433511	ECOG 5103	4,950	HER2 negative N+ or high risk N−	AC [×4]/weekly paclitaxel [×12]/placebo versus AC [×4]/weekly paclitaxel [×12]/BEV versus AC [×4]/weekly paclitaxel [×12]/BEV (followed by BEV alone until 1 year)

Identifiers are from clinicaltrials.gov website.

Abbreviations: *n*: estimated number of patients; N+: lymph nodes positive; N−: lymph nodes negative; CT: chemotherapy; BEV: bevacizumab; TRAST: trastuzumab; FEC: 5-fluorouracil/epirubicin/cyclophosphamide; AC: adriamycin/cyclophosphamide.
